# Association of the *TBK1* mutation p.Ile334Thr with frontotemporal dementia and literature review

**DOI:** 10.1002/mgg3.547

**Published:** 2019-01-22

**Authors:** Huiling Yu, Wenbo Yu, Su‐shan Luo, Yu‐Jie Yang, Feng‐Tao Liu, Yue Zhang, Yan Chen, Yi‐min Sun, Jian‐jun Wu

**Affiliations:** ^1^ Department & Institute of Neurology, Huashan Hospital Fudan University Shanghai China; ^2^ Department of Neurology Jing’an District Center Hospital of Shanghai Shanghai China

**Keywords:** frontotemporal dementia, gene, TBK1, variant

## Abstract

**Background:**

The mutation of TANK‐binding kinase 1 (*TBK1*) gene has been regarded as a causative gene of frontotemporal dementia (FTD)‐amyotrophic lateral sclerosis (ALS) spectrum disease in recent years. So far, more than 70 *TBK1* variants have been identified in patients with FTD‐ALS spectrum.

**Methods:**

We reported a Chinese FTD patient carrying TBK1 p.Ile334Thr variant detected by target sequencing and Sanger sequencing. The patient's clinical materials were collected. The transcription and translation levels of TBK1 mutant were investigated in fibroblast by qPCR and western blot. The effects of TBK1 mutant in inflammation pathway and autophagy were detected by luciferase reporter assay and GST pull‐down assay.

**Results:**

The patient was diagnosed as behavioral variant FTD (bvFTD) and displayed progressively severe cognitive impairment especially in executive function. A pattern of frontotemporal atrophy and hypometabolism was shown through MRI and PET‐CT. In vitro functional experiments of TBK1 p.Ile334Thr variant demonstrated reduced transcription and translation levels, decreased kinase activity but maintenance of interaction with optineurin. The variant was classified as likely pathogenic according to American College of Medical Genetics and Genomics guideline.

**Conclusion:**

We proposed the TBK1 mutation p.Ile334Thr as a likely pathogenic variant in bvFTD which also expanded the clinical spectrum of this variant. It can partially abrogate TBK1 functions and be responsible for FTD‐ALS spectrum diseases through neuroinflammatory pathway.

## 1 INTRODUCTION

Frontotemporal dementia (FTD) is a progressive neurodegenerative syndrome with the clinical manifestations of executive dysfunction, language deficits, and behavior as well as personality changes (Perry & Miller, 2013). It was the second common in young‐onset dementia, accounting for 20% dementia in 45–64 age‐group (Mercy, Hodges, Dawson, Barker, & Brayne, 2008). Its clinical subtypes include behavioral variant FTD (bvFTD) and two primary progressive aphasias: nonfluent aphasia and semantic dementia. Extrapyramidal symptoms and/or amyotrophic lateral sclerosis (ALS) could also be presented in FTD patients (Fujioka & Wszolek, 2011).

Frontotemporal dementia is a syndrome of genetic heterogeneity. Since *MAPT* was first identified as the causative gene of FTD in 1998, an increasing number of pathogenic genes have been reported associated with FTD including *GRN*, *C9orf72*, *VCP*, *SQSTM1*, *CHMP2B, TBK1, OPTN*, *TARDBP*, *CHCHD10*, *UBQLN2,* and *DCTN1 *(Pottier, Ravenscroft, Sanchez‐Contreras, & Rademakers, 2016). Most of them were also the causative genes of ALS except for *GRN* (Mackenzie & Neumann, 2016; Renton et al., 2011; Van Mossevelde, van der Zee, Cruts, & Van Broeckhoven, 2017). Besides, approximately 15% FTD patients develop clinical features of ALS (Lomen‐Hoerth, Anderson, & Miller, 2002) and up to 15% ALS patients finally reach the criteria of FTD, ALS, and FTD are now considered as ALS‐FTD spectrum, other than two separate diseases.

*TBK1* (OMIM:604834) is the causative genes of FTD‐ALS recognized recently based on a large‐scale whole‐exome sequencing study and the following variant analysis (Cirulli et al., 2015; Le Ber et al., 2015). Up till now, more than 70 *TBK1* variants have been reported in ALS, FTD, or FTD‐ALS patients and most were Caucasian (Ahmad, Zhang, Casanova, & Sancho‐Shimizu, 2016). Its encoding protein, tumor necrosis factor receptor‐associated factor NF‐kB activator‐binding kinase 1 (TBK1), was a serine/threonine protein kinase involved in multiple cellular pathways. It has functions in neuroinflammation through NF‐κB pathway and can interact with downstream proteins such as optineurin and p62, which are involved in selective autophagy degradation. In vitro functional assessments showed that mutations of *TBK1* were associated with disruption of inflammation or autophagy pathways for the impairment of optineurin binding or IFN‐β signaling inducement, respectively (Freischmidt et al., 2015).

In this study, we reported a Chinese FTD patient carrying mutation of p.Ile334Thr in *TBK1*. This mutation was firstly reported by Shu et al. in a Chinese ALS patient with no symptoms of FTD (Shu et al., 2016). We also performed the in vitro functional studies to investigate its potential pathogenesis.

## 2 METHODS

### 2.1 Ethical compliance

This research was approved by the Institutional Ethics Committee of Huashan Hospital Fudan University. The study was conducted after receiving written informed consent from the patient.

### 2.2 Clinical materials

The patient came from central China. The clinical materials were collected. Blood biochemical analysis (serum creatine kinase, electrolytes, glucose, retinal and liver function, thyroxine, thyroid‐stimulating hormone, and sedimentation rate) and examinations including cranial magnetic resonance imaging (MRI), positron emission computed tomography (PET/CT), electromyography plus nerve conduction velocity (EMG+NCV), electroencephalogram (EEG), and neuropsychological assessments were carried out.

The diagnosis of bvFTD was made according to the Rascovsky criteria (Rascovsky et al., 2011).

### 2.3 Genetic test

Genomic DNA was extracted from peripheral blood through a standard method (Qiagen, German). A panel containing over 4,000 known virulence genes was performed by target sequencing of the exons. In brief, all exons and their corresponding flanking regions of these genes were selected as target regions. Paired‐end sequencing was performed on Illumina HiSeq X‐ten platform. All variants different from the reference sequence were further screened by allele frequency <1% according to 1,000 Genomes Project (http://www.internationalgenome.org/data), Inhouse database, ESP6500 (evs.gs.washington.edu/EVS/), and ExAC (exac.broadinstitute.org). The synonymous variants were excluded. The phenotypes of the screened genes were compared with the clinical manifestations of the patient, and the inherited modes were considered to further exclude irrelevant genes. Then, the mutations left were confirmed by Sanger sequencing. The databases including Mutation Taster and MUpro were used to predict the pathogenicity of the variants.

The GenBank version was CM000674.1. The NCBI Reference Sequence of *TBK1* gene in this study was NC_000012.11, with a transcript ID of ENST00000331710.5. The NCBI Reference Sequence of *TBK1* mRNA was NM_013254.4, which encodes a protein of 729 amino acids (NP_037386.1). Interpretation of the variants was based on the American College of Medical Genetics and Genomics (ACMG) recommended standards (Richards et al., 2015).

### 2.4 In vitro functional studies

#### 2.4.1 Cell culture and transfection

Fibroblasts were obtained from this patient and healthy controls. Cells were maintained in Dulbecco's modified Eagle's medium (DMEM) supplemented with 20% FBS (Gibco, Carlsbad, CA, USA), nonessential amino acids (Gibco), sodium bicarbonate (Sigma‐Aldrich, St. Louis, MO, USA), and 1% (vol/vol) penicillin/streptomycin/fungizone (Cellgro, Manassas, VA, USA) in an incubator at 37°C under 5% CO_2._

HEK293T cells were cultured in DMEM with 10% FBS in an incubator at 37°C under 5% CO_2_. For transient overexpression, cells were transfected with Lipofectamine 2000 (Life Technologies, Grand Island, NY, USA) according to the manufacturer's instructions.

#### 2.4.2 TBK1 and phosphorylated IRF3 protein analysis

Fibroblasts from both healthy control and the patient were washed twice with phosphate‐buffered saline (PBS) and lysed in RIPA buffer with protease and phosphatase inhibitor cocktails on ice. Proteins were resolved by SDS‐PAGE and transferred to a PVDF membrane (GE Healthcare, Little Chalfont, UK). After being blocked with 5% skim milk, the membrane was then incubated with primary antibodies overnight, followed by HRP‐conjugated secondary antibodies (Santa Cruz Biotechnology, Dallas, TX, USA). Detection was made with a West‐Q Chemiluminescent Substrate Plus Kit (GenDEPOT, Barker, TX, USA).

#### 2.4.3 TBK1 quantitative real‐time polymerase chain reaction (qRT‐PCR)

Total RNA was isolated with RNA isoplus (Takara, Japan) from the fibroblast, and cDNA was synthesized via Toyobo cDNA kit (Toyobo, Japan). Power SYBR Green PCR Master Mix and primers were used to amplifying cDNA. The relative quantities (RQs) of TATA‐binding protein (TBP) were calculated as internal control in the $2^{-\Delta \Delta C_{\text{t}}}$ method. The following primers were used in this study: *TBK1*, 5′‐CGAGATGTGGTGGGTGGAATG‐3′ and 5′‐CACAGACTGTCCATCTTCCCC‐3′; *TBP*, 5′‐CCCATGACTCCCATGACC‐3′ and 5′‐TTTACAACCAAGATTCACTGTGG‐3′.

#### 2.4.4 Plasmids and constructs

N‐terminally GFP‐tagged wild‐type human *TBK1* cDNA was cloned into the pReceiver vector (GeneCopoeia, Rockville, MD, USA). The missense variants (c.1001T>C, p.Ile334Thr) were introduced into wild‐type GFP‐TBK1 using the EZchange™ site‐directed mutagenesis kit (Enzynomics, Daejeon, Korea) according to the manufacturer's protocol. For expression in bacteria, *OPTN* cDNA fragments were amplified by PCR and subcloned into pGEX6P1 (GE Healthcare). All constructs were verified by Sanger sequencing.

#### 2.4.5 GST pull‐down assay

Recombinant GST fusion protein GST‐OPTN was produced in *E. coli* BL‐21 cells. HEK293T cells were transfected with plasmids of wild‐type human *TBK1* and p.Ile334Thr *TBK1* separately. Both cells were lysed in lysis buffer of GST Protein Interaction Pull‐Down Kit (Thermo Scientific™, #21516). Following experiments were performed according to the manufacturer's protocol.

#### 2.4.6 Luciferase reporter assay

For luciferase reporter assays, HEK293T cells were cultured on 24‐well plates and cotransfected with a plasmid encoding an IFN‐β luciferase reporter (NanoLuc luciferase; 500 ng), pGL4.54 (firefly luciferase plasmid; 50 ng), and 1,000 ng of a plasmid‐encoding GFP‐tagged wild‐type or p.Ile334Thr *TBK1*. Transfection was performed with Lipofectamine 2000 (Invitrogen). At 24 hr post‐transfection, the cells were washed with PBS. The levels of luciferase activity (NanoLuc luciferase and firefly luciferase) were measured according to the manufacturer's protocol (Promega, Mannheim, Germany). Three independent experiments were performed using triplicate samples in each experiment.

#### 2.4.7 Antibodies

Anti‐TBK1 (1:1,000; Thermo Fisher Scientific, Rockford, IL, PA5‐17478), anti‐IRF3 (1:1,000; Abcam, MA, #ab25950), anti‐pIRF3 (Ser396) (1:500 Cell Signaling, MA, 4D4G, #4947), anti‐OPTN(1:1,000, Proteintech, 108371‐AP), and anti‐β‐actin (1:10,000; Sigma, #A5060) antibodies were used. The following secondary HRP‐conjugated antibodies were used for immunoblotting: goat anti‐mouse IgG (1:1,000; Santa Cruz, CA, #sc‐2005) and goat anti‐rabbit IgG (1:1,000; Santa Cruz, #sc‐2004).

## 3 RESULTS

### 3.1 Clinical evaluation

The patient was a 38‐year‐old female who referred to Huashan Hospital after a one‐year history of slowness in reaction, changes in personality, slow movement, and stiffness in her extremities under no predisposing cause. She has no past medical history. She had a suspicious family history as her father had “mental problem” and got lost in his thirties. During her last physical examination, two years from disease onset, the patient showed less cooperation, hyperactivity, and communication difficulties. She also showed stereotyped movement which was repeatedly rubbing her left thigh with her left hand several times and then touching her mouse or hair. Forced grasping was induced in her left hand. Her right hand presented stiffness of all fingers and could not perform fine operations. There was no muscle atrophy. Muscle strength and deep tendon reflexes were normal, while the muscle tone of her upper limbs was elevated with the right one more obvious. Bilateral Babinski signs, Hoffmann signs, and palmomental reflex were negative. The patient scored 12 in Neuropsychiatry Inventory Assessment, 39 in Dysexecutive Questionnaire, 33 in Frontal Behavioral Inventory, and 10.5 in Clinical Dementia Rating (sum of box), suggesting severe cognitive impairment particularly in executive dysfunction, attention deficit, and loss of empathy (Table 1).

**Table 1:** Neuropsychological assessments of the FTD patient carrying TBK1 variant of p.Ile334Thr

Test	Score/total score	Cutoff score	Reference
Mini‐mental state examination	19/30^a^	≥24	Strong et al. (2009)
Boston naming test	12/30^a^	≥24	Jefferson et al. (2007)
Stroop test	Fail to Conduct	–	Scarpina and Tagini (2017)
Auditory verbal learning test			
AVLT‐I	5/36^a^	≥12	Crossen and Wiens (1994)
AVLT‐T	6/60^a^	≥23	
Neuropsychiatry inventory	12/36	–	Lai (2014)
Dysexecutive questionnaire	39/80^a^	<10	Pedrero‐Perez et al. (2011)
Frontal behavioral inventory	33/72^a^	<27	Kertesz, Davidson, and Fox (1997)
Clinical dementia rating sum of box	10.5/18^a^	<2.5	O'Bryant et al. (2010)

Note

FTD: frontotemporal dementia.

a Score outside the normal value.

**Figure 1:**
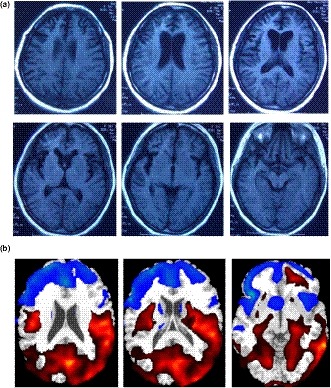
Imaging findings of the patient. (a) Cerebral MRI showed bilateral atrophy of frontal and temporal lobes. (b) FDG‐PET showed severe hypometabolism in frontal temporal areas

Cranial MRI indicated bilateral frontotemporal atrophy (Figure 1a). Her PET‐CT findings also suggested severe bilateral frontotemporal hypometabolism (Figure 1b). Both EMG and EEG were normal. She was diagnosed as bvFTD and donepezil, amantadine and clonidine were prescribed.

### 3.2 Mutation analysis

**Figure 2:**
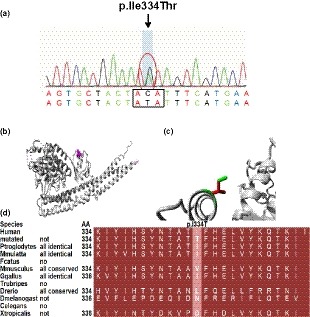
Detection of p.Ile334Thr in *TBK1* (NP_037386.1). (a) Sanger sequencing showed the heterozygous of the mutation. (b) The overview of protein. The protein is colored gray, and the side chain of the mutant residue is colored magenta. (c) Close‐up of the mutation. The protein is colored gray, and both the wild‐type (green) and mutant (red) residue are shown. (d) Conservation among multiple species at position 334

The mean depth of target sequencing of the patient was 247.96X. The percentage of the target region with mean depth over 20X was 99.7%. According to the criteria mentioned above, only one heterozygous mutation of c.1001T>C in *TBK1* was found, leading to a p.Ile334Thr change in exon 9 (Figure 2a). The MAF of the variant TBK1 p.Ile334Thr (12:64878091 T/C) was about C = 2/102,266 in the total population. The variant was not detected in 200 Chinese seniors without medical history of neurological diseases from the community by Sanger sequencing. The evaluation by Mutation Taster (Schwarz, Cooper, Schuelke, & Seelow, 2014) and MUpro (Cheng, Randall, Sweredoski, & Baldi, 2005) suggested the variant be deleterious: Mutation Taster‐Disease causing, with a score of 89; MUpro‐Decrease stability, with a score of −1.85. Furthermore, according to the protein analysis on a website (http://www.cmbi.ru.nl/hope/), the mutant residue became smaller and less hydrophobic than the wild type, which might lead to a possible loss of interaction with other molecules on the surface of the protein (Figure 2b,c). The wild‐type residue was not conserved at this position (Figure 2d).

### 3.3 Expression and function analysis

**Figure 3:**
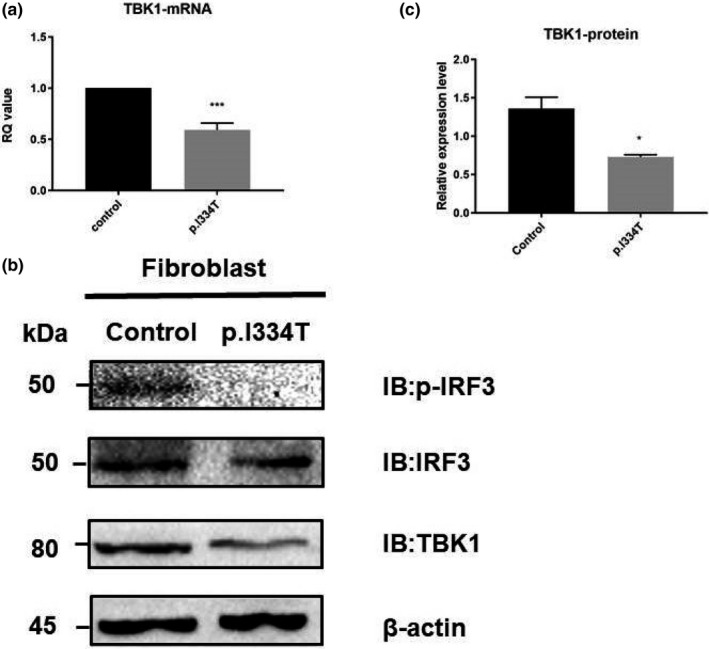
Quantification of mRNA and protein expression level of *TBK1* in patient‐derived fibroblasts. (a) Quantitative RT‐PCR analysis of *TBK1* mRNA level in fibroblasts from healthy control and the patient with p.Ile334Thr mutation. Values are expressed as mean ± *SEM*. ***p < 0.0001; unpaired *t* test. (b) Western blot analysis of TBK1 and phosphorylated IRF3 protein expression level in patient‐derived fibroblasts. β‐actin was used as a loading control. (c) Quantification of TBK1 level. Values are expressed as mean ± *SEM*. *p < 0.05; unpaired *t* test

Compared with control fibroblasts, the mRNA level of *TBK1* in the fibroblasts with p.Ile334Thr variant was decreased by about 50% (Figure 3a), and the same was found in the protein expression (Figure 3b,c).

**Figure 4:**
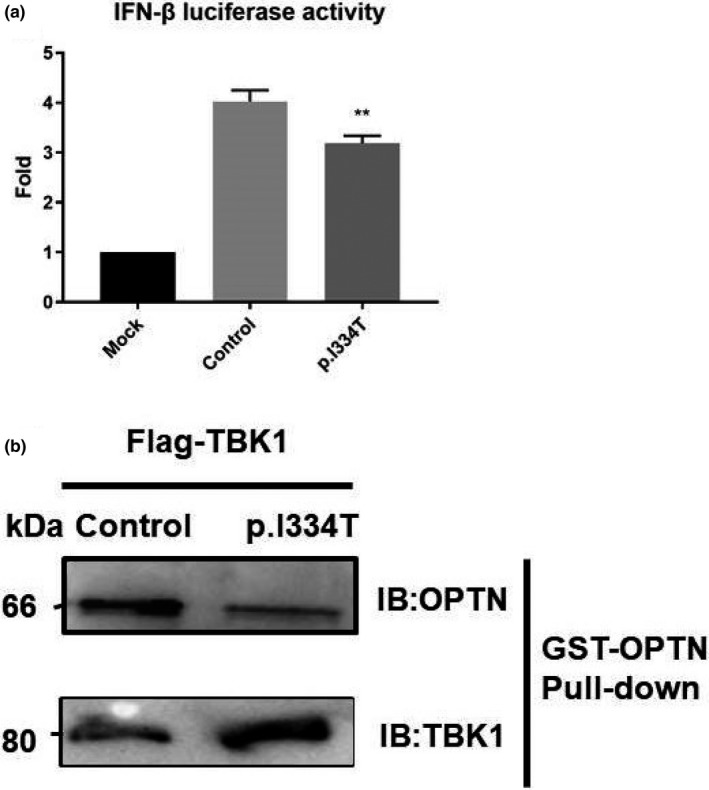
In vitro functional analysis of *TBK1* mutation. (a) Luciferase activity in HEK293T cells cotransfected with an IFN‐β reporter plasmid and a GFP‐tagged wild‐type plasmid or a p.Ile334Thr mutation *TBK1* plasmid for 24 hr. Firefly luciferase activity served as an internal control. Values are expressed as mean ± *SEM*, **p < 0.05. (b) Interaction between TBK1 and its downstream autophagy receptor optineurin. Lysates of a GFP‐tagged wild‐type *TBK1* or p.Ile334Thr from HEK293T cells were incubated with GST‐OPTN. Both cell lysates and bound proteins were detected by western blotting

TBK1 is known to phosphorylate IRF3 and regulate its dimerization and nucleus translocation, which participate significantly in the activation of IFN pathway in inflammation. Thus, the protein level of phosphorylated IRF3 in the fibroblasts of p.Ile334Thr variant was also evaluated and found decreased, suggesting a possible disruption of the downstream IFN pathway, which was then measured by the activation of IFN‐β promoter activity using luciferase reporter assay. Compared with the wild‐type *TBK1*, the p.Ile334Thr variant overexpressed in the HEK293T cells showed approximately 25% reduced IFN‐β signaling (Figure 4a), suggesting a potential disruption of TBK1 anti‐inflammation function.

CCD2 domain of TBK1 phosphorylated its downstream binding protein optineurin. Phosphorylated optineurin then took part in the autophagy. It would be of interest to further test whether autophagy defects are observed in p.Ile334Thr mutant. Here, we examined the direct interactions between TBK1 and optineurin through GST pull‐down assay. We transfected HEK293T cells with GFP‐tagged wild‐type or p.Ile334Thr form of *TBK1*. The whole cell lysates of HEK293T cell were subjected to GST pull‐down assay with GST‐OPTN. Immunoblotting showed that both wild‐type and p.Ile334Thr TBK1 could bind with optineurin (Figure 4b), indicating normal function of TBK1 CCD2 domain.

According to ACMG guideline, the variant was classified as likely pathogenic (Richards et al., 2015).

## 4 DISCUSSION

In the present study, we described an early‐adult onset patient of FTD, carrying a *TBK1* mutation of p.Ile334Thr. The patient displayed severe behavior changes and cognitive impairment especially in executive functions but no evidence of ALS. Further experiments showed reduced transcription and expression level of *TBK1* as well as decreased IFN‐β signaling but normal binding ability with downstream autophagy receptor optineurin, indicating dysfunction of inflammatory pathway with this mutation.

The FTD‐ALS causative gene *TBK1* contributed to about 1.3% ALS, 3%–4% ALS‐FTD, and <1% FTD with TDP‐43 pathology (Nguyen, Van Broeckhoven, & van der Zee, 2018), of whom great clinical heterogeneity was presented. Considering the *TBK1* variants, clinical manifestations of reported mutations in FTD‐ALS spectrum were listed (Table 2). Among the patients with *TBK1* mutations, over 50% patients were diagnosed as ALS, as well as 18% FTD, 14% FTD‐ALS, and 1.3% AD. The age at onset varied from 26 to 86, with a disease course ranging from <1 year to more than 10 years, and approximately 18% patients died within 2 years. The most common initial symptoms included limb weakness, cognitive deficits, bulbar signs such as dysarthria and dysphagia. Interestingly, about half of the patients with *TBK1* mutations were identified with cognitive impairment and one‐thirds with bulbar symptoms. Intrafamilial and interfamilial heterogeneity were also seen in patients carrying the same mutants (Freischmidt et al., 2015). The *TBK1* p.Ile334Thr mutation was first reported in a sporadic ALS (sALS) patient without cognitive impairment (Shu et al., 2016). However, in our case, the patient displayed bvFTD, suggesting this mutation may be obligated to FTD‐ALS spectrum.

**Table 2:** The clinical characteristic of patients harboring TBK1 mutations

Function	Population	Mutation	Diagnosis	Age at onset (y)	Disease duration	Family history	Initial symptoms	Cognitive impairment	MNp	Bulbar sign	Reference
LoF	Japanese	c.1644‐1G>A	ALS	58	>7	Y	Bulbar, lower limbs	N	UMN+LMN	Y	Naruse et al. (2018)
LoF	Italian	c.358+5G>A	ALS	62	>1.4	N	Spinal	N	LMN	NA	Pozzi et al. (2017)
NA	Italian	c.1644–5_1644‐2delAATA	ALS	43	>2.2	N	Spinal	N	UMN+LMN	NA	Pozzi et al. (2017)
LoF	French	IVS18‐2A>G	FTD‐ALS	NA	NA	NA	NA	Y	NA	NA	Le Ber et al. (2015)
LoF	Spanish	p.Gln*2	FTD	56	>4.41	NA	NA	Y	NA	NA	van der Zee et al. (2017)
NA	French	p.Thr4Ala	FTD	NA	NA	NA	NA	Y	NA	NA	Le Ber et al. (2015)
NA	French	p.Gly26Glu	ALS	NA	NA	NA	NA	NA	NA	NA	Le Ber et al. (2015)
LoF	German	p.Lys29Argfs*15	FTD	73	4	NA	NA	Y	NA	NA	van der Zee et al. (2017)
LoF	NA	p.Lys30 Glu76del	ALS	NA	NA	NA	NA	NA	NA	NA	van der Zee et al. (2017)
FMV	German	p.Arg47His^a^	ALS	NA	NA	NA	NA	NA	NA	NA	Freischmidt et al. (2015)
			ALS	NA	NA	NA	NA	NA	NA	NA	
			ALS	NA	NA	NA	Spinal	NA	NA	NA	
LoF	Italian	p.Leu59Phefs*16	ALS	36	4.3	N	Spinal	N	NA	NA	Pozzi et al. (2017)
NA	Chinese	p.Leu62Pro	ALS	47	2.2	N	Limb	N	NA	NA	Shu et al. (2016)
LoF	German	p.Thr77Trpfs*4^a^	ALS	35	>5	Y	Spinal	NA	NA	NA	Freischmidt et al. (2015)
			ALS	58	3	Y	NA	NA	NA	NA	
LoF	Spanish	p.Thr79del	ALS‐FTD	56	1.5	NA	NA	Y	NA	Y	van der Zee et al. (2017)
FMV	Bulgarian	p.Leu94Ser	ALS	44	>10	Y	Spinal	NA	NA	NA	van der Zee et al. (2017)
NA	Chinese	p.Leu94Ser	ALS	NA	3.2	NA	NA	NA	NA	NA	Pang et al. (2017)
LoF	Swedish	p.Val97Phefs*2	ALS	62	<1	Y	NA	NA	NA	NA	van der Zee et al. (2017)
FMV	Swedish	p.Tyr105Cys	ALS	NA	NA	N	NA	NA	NA	NA	Freischmidt et al. (2015)
LoF	Italian	p.Arg117*	FTD	67	7.1	Y	NA	Y	NA	NA	van der Zee et al. (2017)
	American	p.Arg117*^b^	FTD	68	4	N	NA	Y	NA	Y	Pottier et al. (2015)
FMV	Spanish	p.Gly121Asp	ALS	34	>5	NA	Spinal	NA	NA	NA	van der Zee et al. (2017)
LoF	German	p.Arg127*	ALS	70	NA	NA	NA	NA	NA	Y	van der Zee et al. (2017)
FMV	German	p.Arg143Cys	FTD	45	NA	Y	NA	Y	NA	NA	van der Zee et al. (2017)
NA	French	p.Arg143Cys	ALS	NA	NA	NA	NA	NA	NA	NA	Le Ber et al. (2015)
LoF	French	p.Thr156Argfs*6	FTD‐ALS	NA	NA	NA	NA	Y	NA	NA	Le Ber et al. (2015)
LoF	Belgian	p.Asp167del	ALS	60	<1	NA	NA	NA	NA	NA	van der Zee et al. (2017)
LoF	German	p.Tyr185*^a^	ALS	47	>6	Y	Spinal	N	UMN+LMN	N	Freischmidt et al. (2015)
			ALS	37	3	Y	NA	NA	NA	NA	
			ALS	41	6	Y	NA	NA	NA	NA	
			ALS	40	3	Y	NA	NA	NA	NA	
LoF	Italian	p.Asp118Asn	ALS	81	>2.9	N	Spinal	Y	LMN	NA	Pozzi et al. (2017)
FMV	German	p.Arg229Ser	ALS	47	NA	NA	NA	NA	NA	NA	van der Zee et al. (2017)
FMV	Portuguese	p.Gly244Val^c^	FTD‐ALS	41	<2	Y	Bulbar	Y	NA	Y	van der Zee et al. (2017)
FMV	German	p.Ile246Thr	ALS	57	<2	NA	Bulbar	NA	NA	Y	van der Zee et al. (2017)
NA	Belgian	p.Arg271Leu	FTD	80	7	Y	NA	Y	NA	NA	Gijselinck et al. (2015)
LoF	Belgian	p.Gly272_Thr331del	FTD	48	2.4	Y	NA	Y	NA	NA	van der Zee et al. (2017)
NA	Scottish	p.Leu277Val	MND	26	11.8	*N*	limb	NA	NA	NA	Black et al. (2017)
FMV	Italian	p.Lys291Glu	ALS	74	>2.75	*N*	Spinal	Y	LMN	NA	Pozzi et al. (2017)
FMV	Belgian	p.Lys291Glu	FTD	52	<9	Y	NA	Y	NA	NA	van der Zee et al. (2017)
	Belgian	p.Lys291Glu	FTD	60	4	Y	NA	Y	NA	NA	Gijselinck et al. (2015)
FMV	Swedish	p.Ile305Thr	ALS	NA	NA	N	NA	NA	NA	NA	Freischmidt et al. (2015)
FMV	American	p.Leu306Ile	FTD‐ALS	70	2	N	Y	Y	NA	NA	Pottier et al. (2015)
FMV	Swedish	p.Arg308Gln	ALS	37.9	3.5	N	Spinal	N	UMN+LMN	Y	Freischmidt et al. (2015)
NA	French	p.Thr320Ile	ALS	NA	NA	NA	NA	NA	NA	NA	Le Ber et al. (2015)
LoF	Swedish	p.Thr320Glnfs*40^a^	ALS	60.1	1.9	Y	Spinal	NA	UMN+LMN	Y	Freischmidt et al. (2015)
			ALS	NA	NA	Y	NA	NA	NA	NA	
NA	Belgian	p.His322Tyr	ALS	64	2	N	NA	NA	NA	NA	Gijselinck et al. (2015)
NA	Chinese	p.Ile334Thr	ALS	51	4	N	Limb	N	NA	NA	Shu et al. (2016)
NA	Chinese	p.His336Arg	ALS	NA	3.5	NA	NA	NA	NA	NA	Pang et al. (2017)
FMV	Italian	p.Arg357Gln	ALS	36	>1.7	N	Spinal	N	NA	NA	Pozzi et al. (2017)
	Swedish	p.Arg357Gln	ALS	61.1	3	Y	Bulbar	N	UMN+LMN	Y	Freischmidt et al. (2015)
NA	Sardinian	p.Arg384Thr	ALS	NA	1.6	Y	Limb	N	UMN+LMN	Y	Borghero et al. (2016)
NA	Italian	p.Ile397Thr	ALS	65	>5	N	Spinal	Y	UMN+LMN	NA	Pozzi et al. (2017)
LoF	Belgian	p.Ser398Profs*11	ALS	59	>6.25	Y	NA	NA	NA	Y	van der Zee et al. (2017)
NA	Chinese	p.Leu399fs^a^	ALS	60	5.7	Y	Right arm	Y	LMN	*N*	Williams et al. (2015)
			ALS	73	8	Y	Right leg	NA	UMN	NA	
NA	American	p.Lys401Glu	AD	80	10	NA	Y	Y	NA	NA	Pottier et al. (2015)
LoF	Swedish	p.Ala417*	FTD	68	2.25	Y	NA	Y	NA	NA	van der Zee et al. (2017)
	Swedish	p.Ala417*^a^	ALS	65	>7.3	Y	Spinal	Y	UMN+LMN	Y	Freischmidt et al. (2015)
			FTD	NA	NA	Y	Spinal	Y	NA	Y	
			ALS	56	2	Y	Spinal	N	UMN+LMN	Y	
	Swedish	p.Ala417*	ALS	62.2	1.7	N	Bulbar	N	UMN+LMN	Y	Freischmidt et al. (2015)
	Swedish	p.Ala417*	FTD‐ALS	61.9	6.3	N	Spinal	Y	UMN+LMN	Y	Freischmidt et al. (2015)
	Swedish	p.Ala417*	ALS	64.7	2.7	N	Bulbar	N	UMN+LMN	Y	Freischmidt et al. (2015)
FMV	Portuguese	p.Ile418Val	FTD	53	<1	Y	NA	Y	NA	NA	van der Zee et al. (2017)
NA	Italian	p.Tyr424Asp	ALS	68	>0.75	NA	Spinal	Y	UMN+LMN	Y	Piaceri et al. (2018)
LoF	French	p.Arg440X	ALS	NA	NA	Y	NA	NA	NA	NA	Le Ber et al. (2015)
	French	p.Arg440X^a^	ALS	47	13.0	Y	Spinal	N	UMN+LMN	Y	Freischmidt et al. (2015)
			FTD‐ALS	58	3.0	Y	Spinal	Y	UMN	NA	
			ALS	73	4.0	Y	Spinal	NA	UMN	Y	
			ALS			Y	NA	NA	NA	NA	
			FTD	60	16.0	Y	NA	Y	NA	NA	
NA	Sardinian	p.Arg444Gln	FTD‐ALS	72	>0.8	N	Cognition impairment	Y	UMN+LMN	Y	Borghero et al. (2016)
LoF	Chinese	p.Arg444X	FTD‐ALS	55	4	N	Cognition impairment+Limb	Y	NA	Y	Tsai et al. (2016)
LoF	Spanish	p.Trp445*	FTD+CBS	78	>6	NA	NA	Y	NA	NA	van der Zee et al. (2017)
LoF	Danish	p.Ile450Lysfs*15^a^	ALS	56.9	1.9	Y	Spinal	N	UMN+LMN	Y	Freischmidt et al. (2015)
			ALS+D	71	3	Y	Spina	Y	NA	Y	
			ALS	77	>0.7	Y	Bulbar	N	UMN+LMN	Y	
			ALS	71	2	Y	Spinal	N	NA	Y	
			ALS	51	2.2	Y	Spinal	N	UMN+LMN	*N*	
	Swedish	p.Ile450Lysfs*15^a^	ALS	54.9	>3.2	Y	Spinal	N	UMN+LMN	Y	Freischmidt et al. (2015)
			ALS	54	3	Y	Spinal	N	UMN+LMN	Y	
LoF	German	p.Thr462Lysfs*3	ALS+D	74	0.9	Y	NA	Y	LMN	NA	van der Zee et al. (2017)
NA	German	p.Thr462Lysfs^†^	FTD‐ALS	75	<1	Y	NA	Y	NA	Y	Schonecker et al. (2016)
			FTD‐ALS	77	<1	Y	NA	Y	NA	Y	
LoF	Korean	p.Ile472Serfs*8	ALS	53	>3.8	N	Bulbar	N	UMN	Y	Kim et al. (2017)
LoF	Scottish	p.Glu476fs^c^	MND	44	2.83	Y	Limb	NA	NA	NA	Black et al. (2017)
LoF	Swedish	p.Val479Glufs*4^a^	FTD‐ALS	64.7	1.2	Y	Spinal	Y	UMN+LMN	Y	Freischmidt et al. (2015)
			FTD‐ALS	58	3.0	Y	Spinal	Y	NA	NA	
LoF	French	p.Tyr482X	FTD‐ALS	NA	NA	NA	NA	Y	NA	NA	Le Ber et al. (2015)
NA	Belgian	p.Ile515Thr	ALS	59	>10	NA	NA	NA	NA	NA	Gijselinck et al. (2015)
LoF	Belgian	p.Ser518Leufs*32	ALS	64	0.5	Y	NA	NA	NA	Y	van der Zee et al. (2017)
NA	Belgian	p.Ala535Thr	FTD	52	10	Y	NA	Y	NA	NA	Gijselinck et al. (2015)
FMV	Portuguese	p.Met559Arg	ALS	60	6	Y	Spinal	N	UMN+LMN	NA	Freischmidt et al. (2015)
FMV	Swedish	p.Ala571Val	ALS	66.6	1.9	N	Bulbar	N	UMN+LMN	Y	Freischmidt et al. (2015)
NA	Spanish	p.Arg573Gly^a^	FTD	65	13	NA	NA	NA	NA	NA	Gomez‐Tortosa et al. (2017)
			FTD	61	6	Y	NA	NA	NA	NA	
			Dysarthria	60	17	Y	Memory deficit	Y	N	Y	
			D	65	4	Y	Memory deficit	Y	N	Y	
			PLS	66	9	Y	Bulbar	NA	UMN	Y	
			PLS	61	6	Y	Bulbar	NA	N	Y	
FMV	Swedish	p.Met598Val	ALS	61.8	>2.5	N	Bulbar	N	UMN+LMN	Y	Freischmidt et al. (2015)
LoF	Belgian	p.Glu643del^a^	FTD‐ALS	62	11.3	Y	NA	Y	NA	NA	Gijselinck et al. (2015) and van der Zee et al. (2017)
			D	71	10	Y	NA	Y	NA	NA	
			D	86	4	Y	Spinal	Y	NA	NA	
			ALS	69	3	Y	NA	NA	NA	NA	
			FTD	69	6	Y	NA	Y	NA	NA	
			D	70	3	Y	NA	Y	NA	NA	
			FTD	63	>3	Y	NA	Y	NA	NA	
			FTD	66	6	Y	NA	Y	NA	NA	
			FTD	61	13	Y	NA	Y	NA	NA	
			D	63	6	Y	NA	Y	NA	NA	
			D	73	11	NA	NA	Y	NA	NA	
			D	82	4	NA	NA	Y	NA	NA	
			ALS	63	1	NA	NA	NA	NA	NA	
	German	p.Glu643del	ALS	NA	NA	N	NA	NA	NA	NA	Freischmidt et al. (2015)
	German	p.Glu643del	ALS	NA	NA	N	Spinal	NA	UMN	Y	Freischmidt et al. (2015)
	Belgian	p.Glu643del	FTD	64	>9.1	Y	NA	Y	NA	NA	van der Zee et al. (2017)
	Belgian	p.Glu643del	ALS	51	1.7	Y	NA	NA	NA	Y	van der Zee et al. (2017)
	Belgian	p.Glu643del	ALS	63	3	NA	NA	NA	NA	NA	van der Zee et al. (2017)
	Belgian	p.Glu643del	FTD	70	3.5	Y	NA	Y	NA	NA	van der Zee et al. (2017)
	Belgian	p.Glu643del	FTD	69	>8.3	NA	NA	Y	NA	NA	van der Zee et al. (2017)
	Belgian	p.Glu643del	FTD	70	> 6	N	NA	Y	NA	NA	Gijselinck et al. (2015)
	Belgian	p.Glu643del	FTD	69	> 7	N	NA	Y	NA	NA	Gijselinck et al. (2015)
	Belgian	p.Glu643del	ALS	41	<1	N	Bulbar	NA	NA	Y	Gijselinck et al. (2015)
LoF	French	p.Gln655X	FTD‐ALS	NA	NA	NA	NA	Y	NA	NA	Le Ber et al. (2015)
NA	French	p.Met662Thr	FTD‐ALS	NA	NA	NA	NA	Y	NA	NA	Le Ber et al. (2015)
NA	Sardinian	p.Met690fs^d^	ALS	66	>8.6	N	Limb	N	LMN	Y	Borghero et al. (2016)
LoF	Danish	p.690‐713del^a^	ALS	62.7	3.8	Y	Spinal	N	UMN+LMN	Y	Freischmidt et al. (2015)
			ALS	52	2.0	Y	Spinal	Y	NA	Y	
			ALS‐FTD	74	1.2	Y	Spinal	Y	UMN+LMN	Y	
			mono paresis	52	>0.5	Y	Spinal	N	LMN	N	
			possible FTD	NA	NA	Y	Spinal	Y	NA	NA	
	Swedish	p.690‐713del^a^	ALS	64	2.2	Y	Bulbar	N	UMN+LMN	Y	Freischmidt et al. (2015)
			FTD‐ALS	65	5.0	Y	Spinal	Y	UMN+LMN	Y	
			possible FTD	78	3.0	Y	NA	Y	NA	NA	
			D	65	2.0	Y	NA	Y	NA	NA	
FMV	American	p.Glu696Lys	AD	78	6.0	NA	NA	Y	NA	Y	Pottier et al. (2015)
	Swedish	p.Glu696Lys	ALS‐FTD	61	1.3	N	Bulbar	Y	UMN+LMN	Y	Freischmidt et al. (2015)
	Swedish	p.Glu696Lys^a^	ALS	65.5	1.7	Y	Bulbar	N	UMN+LMN	Y	Freischmidt et al. (2015)
			ALS	NA		Y	Bulbar	NA	NA	Y	
LoF	Scottish	p.Ala705fs^e^	MND	46	1.91	N	Bulbar	NA	NA	Y	Black et al. (2017)

Note

NCBI Reference Sequence of TBK1 protein: NP_037386.1.

AD: Alzheimer's disease; ALS: amyotrophic lateral sclerosis; CBS: corticobasal syndrome; D:dementia; FMV: functional missense variant; FTD: frontotemporal dementia; LoF: loss of function; MND: motor neuron disease; *N*: no; NA: not assessed; PLS: primary lateral sclerosis; Y: yes.

^a^Members of a family carrying the same TBK1 variant. ^b^Co‐exist with OPTN mutation p.Gly538Glufs*27. ^c^Co‐exist with C9orf72 repeat expansion. ^d^Co‐exist with SQSTM1 mutation p.Arg33Val. ^e^Co‐exist with OPTN mutation p.Gln314Leu.

Although the Caucasian accounts for most of *TBK1* mutants, mutations in *TBK1* also accounted for sALS patients of Asian population. The frequency of pathogenic or potentially toxic variants of *TBK1* in Korean and Japanese sALS patients was estimated to be 0.8% and 1.26% separately (Kim et al., 2017; Tohnai et al., 2018). Besides, five missense variants of *TBK1* have been reported in ALS patients of Chinese population so far, among which only the patient with p.Arg444X displayed FTD‐ALS, while the others reported no cognitive dysfunction (Tsai et al., 2016). In addition, one frameshift mutation (p.Leu339fs) of Chinese origin was also found in an Australian cohort study of ALS (Williams et al., 2015). Taken together, although infrequent, *TBK1* mutants can still be considered responsible for ALS‐FTD spectrum in Chinese patients.

*TBK1* encodes a multimeric kinase regulating multiple cellular pathways, especially inflammation and autophagy. It consists of 4 domains: a kinase domain (KD), an ubiquitin‐like domain (ULD), a helical scaffold dimerization domain (SDD), and a C‐terminal domain (Larabi et al., 2013). *TBK1* phosphorylates transcription factor IRF3 in the C‐terminal regulatory domain, leading to its dimeration. Phosphorylated IRF3 then translocates to the nucleus, initiating the transcription of type I interferon genes. The ULD is a regulatory component of *TBK1* which controls homodimerization, kinase activation, and interactions with other molecules in the IFN pathway (Li et al., 2012). The p.Ile334Thr mutation locates in the ULD region. Via in vitro functional study, we found reduced expression level of *TBK1* mRNA and protein, as well as disruption in the luciferase activity of IFN‐β, the same as p.Arg357Gln variant which is also located in the ULD region (Freischmidt et al., 2015). Our results implied that *TBK1* p.Ile334Thr mutation disturbed TBK1 homodimerization and kinase activation, resulting in impairment of downstream neuroinflammatory pathways.

CCD2 mutations abrogated optineurin binding, which is necessary for selective autophagy, but not phosphorylation and activation of IRF3. TBK1 interacts with autophagy receptor proteins which can bind with ubiquitinated cargos and transport them into autophagosomes. In addition, TBK1 also participates in the process of transferring autophagosomes into hydrolytic autophagolysosome, leading to the final degradation of autophagy receptors and their target cargo (Freischmidt et al., 2015). In our work, p.Ile334Thr *TBK1* seemed to have normal function in selective autophagy as its maintenance of the binding ability with optineurin through GST pull‐down assay. However, further analysis such as autophagy flux assays should be performed so as to unequivocally identify the full function of *TBK1* p.Ile334Thr in autophagy.

Although aberrant autophagy is considered as a main cause of patients with *TBK1* variants predisposed to FTD‐ALS spectrum, evidence of neuroinflammation in *TBK1* mutations also displayed its significance. Impaired migration of T cells was observed in *TBK1* knockout mice, suggesting a decreased T‐cell regulation which led to less stabilization of glial cell and proinflammatory cytokines (Komine & Yamanaka, 2015). On the other hand, multiform effects of type I IFNs and cytokines have been demonstrated in neuron survival, suggesting IFN impairment in *TBK1* mutations may also lead to the pathogenesis of FTD‐ALS spectrum. In addition, possible relationship between IFN pathways and autophagy in disease has been considered (Watson et al., 2015), implying that optineurin–TBK1 complexes may regulate IRF3–IFN responses. Whether such IFN impairment might also contribute to FTD pathogenesis is a possibility that remains unexplored.

There exist some limitations in this study. First, limited by the number of patients, we only detected one patient with p.Ile334Thr of TBK1. Although we detect that the mutation is likely pathogenic and can partially disturb neuroinflammatory pathway without interfering with the autophagy function of TBK1, additional patients with this mutation need to be identified with functional work. On the other hand, the decreased level of IFN‐β signaling of this mutation indicated that besides the disturbance of neuroinflammatory pathway, there may still be some unknown pathogenic mechanism under this mutation that we have not yet discovered.

## 5 CONCLUSION

In this article, we reported a *TBK1* mutation of p.Ile334Thr in a Chinese bvFTD patient and classified it as likely pathogenic. Clinical evaluation confirmed the diagnosis of bvFTD, and functional analysis of the mutation demonstrated partially dysfunction of protein expression and activity of *TBK1*. Whether dysfunction of neuroinflammatory pathways reveals a general feature of p.Ile334Thr mutation of *TBK1* remains to be explored. Further study will focus on the pathogenesis of this mutation on FTD and its relationship with ALS.

## CONFLICT OF INTEREST

The authors report no conflicts of interest.
